# Molecular Phylogeny and Predicted 3D Structure of Plant *beta*-D-*N*-Acetylhexosaminidase

**DOI:** 10.1155/2014/186029

**Published:** 2014-07-20

**Authors:** Md. Anowar Hossain, Hairul Azman Roslan

**Affiliations:** ^1^Department of Biochemistry and Molecular Biology, University of Rajshahi, Rajshahi 6205, Bangladesh; ^2^Genetic Engineering Laboratory, Department of Molecular Biology, Faculty of Resource Science and Technology, Universiti Malaysia Sarawak, 94300 Kota Samarahan, Sarawak, Malaysia

## Abstract

*beta*-D-*N*-Acetylhexosaminidase, a family 20 glycosyl hydrolase, catalyzes the removal of *β*-1,4-linked *N*-acetylhexosamine residues from oligosaccharides and their conjugates. We constructed phylogenetic tree of *β*-hexosaminidases to analyze the evolutionary history and predicted functions of plant hexosaminidases. Phylogenetic analysis reveals the complex history of evolution of plant *β*-hexosaminidase that can be described by gene duplication events. The 3D structure of tomato *β*-hexosaminidase (*β*-Hex-Sl) was predicted by homology modeling using 1now as a template. Structural conformity studies of the best fit model showed that more than 98% of the residues lie inside the favoured and allowed regions where only 0.9% lie in the unfavourable region. Predicted 3D structure contains 531 amino acids residues with glycosyl hydrolase20b domain-I and glycosyl hydrolase20 superfamily domain-II including the (*β*/*α*)_8_ barrel in the central part. The *α* and *β* contents of the modeled structure were found to be 33.3% and 12.2%, respectively. Eleven amino acids were found to be involved in ligand-binding site; Asp(330) and Glu(331) could play important roles in enzyme-catalyzed reactions. The predicted model provides a structural framework that can act as a guide to develop a hypothesis for *β*-Hex-Sl mutagenesis experiments for exploring the functions of this class of enzymes in plant kingdom.

## 1. Introduction

As a part of the study to elucidate the role of free *N*-glycans and de-*N*-glycosylation mechanism working in plants, we have already characterized the PNGase, ENGase, *α*-mannosidase and *β*-hexosaminidase at molecular level [1–3]. The *β*-D-*N*-acetylhexosaminidase (EC 3.2.1.52), a member of the glycosyl hydrolase family 20 (GH20), is an enzyme that hydrolyses nonreducing terminal monosaccharide residues of *β*-*N*-acetylgalactosaminides and *β*-*N*-acetylglucosaminides. It is widely distributed among the animals, insects, plants, fungus, and bacteria. Mammal lysosomal *β*-*N*-acetyl-D hexosaminidases are mainly responsible for glycoconjugate degradation in lysosome. HexA is a heterodimer of subunits *α* (encoded by the gene HexA) and *β* (encoded by the gene HexB), whereas HexB is a homodimer of *β* subunits. The subunits arose through a gene duplication event and the primary sequences are approximately 60% identical. Mutational defects that cause *β*-hexosaminidase-A and B deficiency are responsible for Sandhoff and the Tay-Sachs diseases, respectively [4]. Recently, it has been reported that *β*-hexosaminidase is a surrogate marker for renal function in autosomal dominant polycystic kidney disease [5]. In insects, it has been postulated to have specialized physiological functions, including posttranslational modification of *N*-glycans, degradation of glycoconjugates, and egg-sperm recognition, suggesting that these enzymes have rather versatile physiological functions in the growth and development of insects [6]. Mammal *β*-*N*-acetyl-D-hexosaminidases have been shown to be important for egg-sperm recognition [7], and the enzymes from* Drosophila melanogaster* sperm membrane also participate in the same process [8]. A fungal *β*-*N*-acetyl-D-hexosaminidases has been expressed, characterized, and crystallized from* Aspergillus oryzae,* which has sequence similarity to bacterial and human enzymes ranges from 42% to 49% [9].

Recently, plant *β*-*N*-acetyl-D-hexosaminidases has gained a lot of attention due to its presence in the ripening stages [2]. It has also been shown that the tomato fruit shelf life can be enhanced by the suppression of *N*-glycan processing/degrading enzymes [10]. Plant glycoproteins contain substantial amounts of paucimannosidic *N*-glycans lacking terminal GlcNAc residues at their nonreducing ends. It has been proposed that this is due to the action of *β*-hexosaminidases during late stages of *N*-glycan processing or in the course of *N*-glycan turnover [11]. Although several *β*-hexosaminidases have been reported from various parts of plants such as leaves, fruits, and seeds [10–13], their physiological functions in plant biology are not yet fully understood. To elucidate the exact roles of this enzyme in plant kingdom, it is desirable to know about properties and behavior of the phylogenetically related enzymes from different species and their molecular evolutions. However, little is known about the phylogenetics and evolution of plant *β*-hexosaminidases.

So far eight crystal structures of GH20 *β*-*N*-acetyl-D-hexosaminidases have been reported including two humans, one insect, and six bacterial enzymes. Both the human HexA and HexB are the *β*-*N*-acetyl-D-hexosaminidases that degrade glycoconjugate in the lysosome [4, 14]. OfHex1, the enzyme from the Asian corn borer* Ostrinia furnacalis* (one of the most destructive pests), has been reported to function merely in chitin degradation [6]. The bacterial enzymes include SpHex and SmCHB, which are found in the chitinolytic bacteria* Streptomyces plicatus* and* Serratia marcescens*, respectively [15–17]. AaDspB, which is isolated from* Aggregatibacter actinomycetemcomitans*, is involved in the degradation of biofilm (polymeric *β*-1,6-linked GlcNAc) [18]. The enzyme, PsHex from* Paenibacillus sp*. TS12, can efficiently degrade various glycosphingolipids [19]. PgGcnA, the enzyme found in the endocarditis pathogen,* Streptococcus gordonii*, is involved in the release of dietary carbohydrates [20]. Recently, it has been found that a novel *β*-*N*-acetylhexosaminidase, StrH protein from* Streptococcus pneumoniae R6,* is involved in the catalytic specificity towards the *β* (1,2)-linked *β*-*N*-acetylglucosides and key residues in the active site are Trp-443 and Tyr-482 [21]. Thus, it is interesting to know how these enzymes could carry out their specialized functions in terms of their structural features. To our knowledge, no crystal structure of plant *β*-*N*-acetyl-D-hexosaminidase has yet been reported. Therefore, comparative homology modeling of tomato *β*-*N*-acetyl-D-hexosaminidase is desirable to elucidate the functional prediction, active site information, and mechanism of action.

In the present work, first we identified the 83 homologous sequences of *β*-*N*-acetyl-D-hexosaminidase in GenBank by the NCBI BLAST-PSI search. We did multiple sequence alignments and reconstructed the phylogenetic tree. Secondly, in order to initiate structural studies of this enzyme, we performed sequence alignment and 3D-structure homology modeling and constructed a molecular model of this enzyme and of its complex with the natural substrate. We also performed molecular docking of the enzymes and predicted the active site residues responsible for catalytic activity. The predicted 3D structural information will be useful to study the site-directed mutagenesis wet lab experiments as well as the physiological functions of tomato *β*-*N*-acetyl-D-hexosaminidase in the plant kingdom.

## 2. Material and Methods

### 2.1. Data Retrieval

In this study, we retrieved all of the sequences from the National Center for Biotechnology Information (NCBI) GenBank database as described by Gonzalez and Jordan [22]. Shortly, an initial dataset of the previously published and functionally characterized *β*-*N*-acetyl-D-hexosaminidase amino acid sequences was retrieved manually from Entrez (http://www.ncbi.nlm.nih.gov/entrez). The representative sequences including the *β*-Hex-Sl were isolated from a wide phylogenetic range of eukaryotes and prokaryotes, which possessed a variety of biochemical activities. A CD-Hit clustering program was used to group these sequences by amino acid identities into clusters [23]. Divergent *β*-*N*-acetyl-D-hexosaminidase amino acid sequences with representatives from each cluster were used as queries in a series of PSI-BLAST (Position-Specific Iterated BLAST) searches of the protein database throughout all organisms at NCBI [24]. The representative sequences were* Solanum lycopersicum beta*-hexosaminidase sequence [gi:350540008],* Arabidopsis thaliana* AtHex1[gi:30694211],* Homo sapiens* protein sequences HexA[gi:4261632]* and* HexB[gi:867691]*, Drosophila melanogaster* hexosaminidase sequences Hexo1[gi:17647501]* and* Hexo2[gi:17933586],* Aspergillus oryzae* HexA[gi:169766420], and* Streptomyces plicatus* HexA[gi:13786695]. We chose the sequences from BLAST results based on the high similarities of amino acids (>60% identities) with the query representative sequences. The picked sequences were checked manually to exclude incomplete and redundant sequences. For the feature analysis and construction of phylogenetic tree we took a total of 83 sequences, which are already characterized as predicted or true *β*-*N*-acetyl-D-hexosaminidase from the GenBank, to reduce computational burden. An archea sequence was also retrieved from GenBank that was used as an outgroup in the construction of phylogenetic tree.

### 2.2. Multiple Sequence Alignments and Construction of Phylogenetic Tree

MUSCLE program [25] was used to align all 83 amino acid sequences of *β*-*N*-acetyl-D-hexosaminidases and the alignments were checked manually. Unambiguously aligned regions were identified using GBlocks program [26]. The phylogenetic relationships between the genes were analyzed using the maximum-likelihood (ML) method. For the ML analyses, we used the PROTML program of PHYLIP version 3.6 [27].

We employed the WAG model of amino acid substitution with gamma distribution site rate and invariable site category for phylogenetic analysis [28]. All indels were counted as missing. We performed ten random sequence addition searches using the J option and global branch swapping using the G option to isolate the ML tree with the best log likelihood. In addition, we performed bootstrap analysis with 100 replications.

### 2.3. Comparative Homology Modeling of Tomato *β*-*N*-Acetyl-D-hexosaminidase

Amino acid sequence of* Solanum lycopersicum β*-*N*-acetyl-D-hexosaminidase (*β*-hex-Sl) composed of 575 residues was retrieved from NCBI GenBank (GI: 350540008 and Accession no. NP_001234608.1). The SWISS-MODEL web server [29] was used to identify the template structure, 1now, and also used for homology modeling. The online ModWeb Comparative Modeling Server version SVN.r1340:1348M and I-TASSER [30] were also used for further modeling to compare which is the most correct model. The DFire [31], QMEAN [32], PROCHECK [33], WHAT_CHECK [34], and VERIFY_3D [35] methods and ModEval model evaluation server [36] were used to check the validity of the modeled structures. UCSF Chimera and Swiss-PdbViewer were used to view the models and images preparation. The COFACTOR, a structure-based method for biological function annotation of protein molecules, was used to identify the functional insights including ligand-binding site, gene-ontology terms, and enzyme classification [37–39].

## 3. Results and Discussion

### 3.1. Sequence Analysis of *β*-Hex-Sl

**Figure 1:**
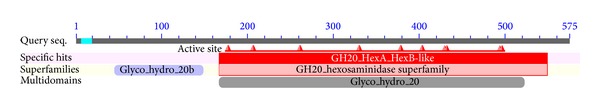
Conserved domains for tomato *β*-hexosaminidase, analyzed using Conserved Domain Database search in NCBI-BLAST.

**Figure 2:**
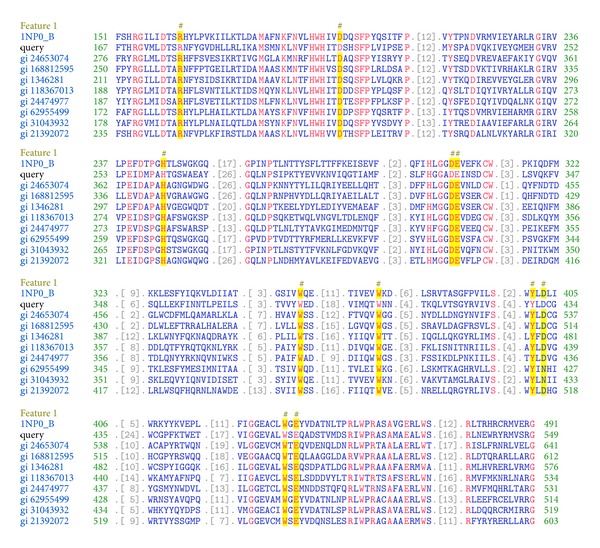
Sequence alignment of *β*-Hex-Sl with nine other sequences by CD search. The amino acid residues Arg(178), Asp(207), His(261), Asp(330), Glu(331), Trp(378), Trp(404), Tyr(430), Asp(432), Trp(494), and Glu(496) were predicted to be responsible for the activity of *β*-Hex-S. The conserved amino acids are shown as yellow color.

The *β*-Hex-Sl protein sequence was analyzed by NCBI CD-search tool (CDD V3.0-44354 PSSMs) to identify the conserved domains (CD). The sequence contains a Glyco_hydro_20b (46~149 aa), GH20_HexA_HexB-like domain (167~549 aa), and a glycosyl hydrolase family 20, catalytic domain (167~522 aa) belongs to the GH20_hexosaminidase superfamily proteins (Figure 1). Based on CD database available and three-dimensional structure-activity relationship, the amino acid residues Arg(178), Asp(207), His(261), Asp(330), Glu(331), Trp(378), Trp(404), Tyr(430), Asp(432), Trp(494), and Glu(496) were predicted to be present in the active site of *β*-Hex-Sl with other sequences (Figure 2). The online tool NetNGlyc 1.0 server was used to identify the *N*-glycosylation site present in the protein sequence. The predicted *N*-glycosylation sites were position at 50 (NFTI), 86 (NLTS), 112 (NESY), 151 (NPTR), 299 (NPSI), 350 (NGTL), 362 (NNTL), 372 (NRTV), 390 (NPSL), 409 (NNTK), and 441 (NDSR) (data not shown). The software SignalP 4.1 server was used to predict the signal peptide cleavage site that was found to be in between positions 23 and 24 in the amino acid sequence.

### 3.2. Phylogenetic Analysis of *β*-Hexosaminidase Sequences

**Figure 3:**
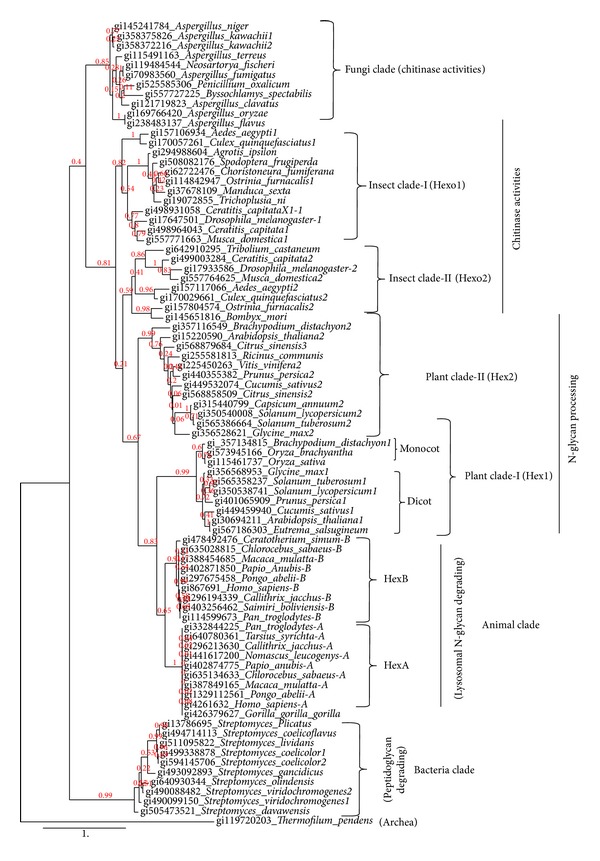
The phylogenetic tree based on* beta*-hexosaminidase amino acid sequences obtained by the maximum likelihood method.* Thermofilum* (Archea) was used as an outgroup to reconstruct the phylogenetic tree. The percentage of replicate trees in which the associated taxa clustered together in the bootstrap test (100 replicates) is shown next to the branches. All analyses were performed with the WAG amino acid substitution model and 1 invariable and 4 gamma distributed site rate categories. Detailed information about the sequences is shown in Table 1.

In order to know the evolutional history and properties of plant* beta*-hexosaminidases, we reconstructed the phylogenetic tree. We aimed to collect the sequence data of the* beta*-hexosaminidases from a wide range of organisms so that we could get a lot of information including their physicochemical, structural, and biological functions. A total of 83 amino acid sequences were retrieved from the GenBank database by previously characterized representative sequences. These sequences used in the analysis include 23 experimentally characterized *β*-*N*-acetyl-D-hexosaminidase enzymes as well as 60 novel predicted or putative *β*-*N*-acetyl-D-hexosaminidase sequences (Table 1). MUSCLE program was also used to align the sequences, whereas maximum likelihood method was used in phylogenetic reconstruction. Our phylogenetic analysis shows that *β*-*N*-acetyl-D-hexosaminidases are widely distributed among plant, animal, insects, fungi, and bacteria, belonging to the glycosyl hydrolase 20 superfamily (Figure 3). It reveals the complex history of evolution of *β*-*N*-acetyl-D-hexosaminidases that can be described by multiple gene duplication events.

Eukaryotic *β*-hexosaminidases might be originated from common bacterial ancestor through multiple gene duplications. Bacteria and fungi clades mostly contain one gene for hexosaminidase in each species albeit few have two genes. Bacteria clade consists of *β*-hexosaminidases that have the peptidoglycan degradation and chitinolytic activities. Those bacterial species, which contain two genes of hexaminidases, might acquire their last copies either by horizontal gene transfer or gene duplication. Fungi sequences clearly showed its own clade and only few species have more than one gene and might be originated either lineage specific mutation and/or gene duplication. Insects clade-I and clade II and plants clade-I and II also contain at least one hexosaminidase gene in each species. Insects (I and II) clades hexosaminidases are chitinolytic enzymes, which separately form paraphylactic groups that could be evolved by gene duplication. Plants clade-I and clade-II also constitute paraphylactic group and also split into monocotyledons and dicotyledons that have functional divergences. Plant *β*-hexosaminidases are involved in *N*-glycan processing of cell walls. Animal clade clearly splits into two clades, A and B, that contain the isoenzymes, HexA and HexB, respectively.

Gene duplication is considered a major driving force for evolution of genetic novelty, thereby facilitating functional divergence and organismal diversity, including the process of speciation. It can be generated by several mechanisms, including tandem duplication, transposition, and large-scale duplication (e.g., segmental/whole genome duplication (WGD)). Also, segmental duplications (SDs) are increasingly recognized as frequent phenomena, especially in primate genomes; for example, approximately 5% of the human genome consists of duplicated segments [40]. More than 300 gene duplication events have been detected by phylogenetic analysis of plant, animal, and fungi before the separation of three major eukaryotic lineages [41]. Specifically, copy numbers for genes with highly conserved functions seem to be more stable than the number of genes with more divergent functions.* beta*-Hexosaminidases from each kingdom (plant, animal, and insect) are separated into two clades (clusters) and each clade contains at least one member. Human genome data analysis showed that both genes, HexA and HexB, are located in different locus in the chromosomes-15q23-24 and -5q13, respectively. They are originated by gene duplication [42]. Most of the higher eukaryotes contain two or more genes for the hexosaminidases. For example,* Arabidopsis thaliana* contains Hex1, Hex2, and Hex3 [11]. Likewise* Drosophila melanogaster* has three genes, Hexo1, Hexo2, and fdl for hexosaminidase isoenzymes [8]. Even these proteins are also located in different organelles. It has been reported that some legume species have at least two Adh gene loci and resulted from relatively ancient duplication events [43]. From the accumulated evidences and phylogenetic topology, it can be speculated that eukaryotic hexosaminidases might be originated by multiple gene duplication, although more experimental evidences are required to establish our hypothesis.

Most of the prokaryotic and eukaryotic *β*-hexosaminidases reported so far play an important physiological role in chitin recycling, a structural components of cell walls [6, 17, 44]. Plant *β*-hexosaminidases have been investigated in a variety of tissues including seeds and leaves suggesting a role in the storage of glycoproteins [45–47]. They have also been proposed to be involved in plants defense mechanisms and reported as chitin-degrading enzymes [46, 48]. A molecular study of Arabidopsis *β*-hexosaminidases has shown that HEXO1 participates in *N*-glycan trimming in the vacuole, whereas HEXO2 and/or HEXO3 could be responsible for the processing of *N*-glycans present on secretory glycoproteins [11]. The *β*-Hex is also present at high levels during the ripening of many fruits, including the climacteric fruit tomato [49] and mango [50]. Recently, it has been reported that suppression of *N*-glycan processing enzymes increases the shelf life of tomato fruits and capsicum [10, 51]. The *β*-Hex, a cell wall enzyme, cleaves the terminal *N*-acetyl-D-hexosamine residues and generates the paucimannosidic *N*-glycans present in most plant glycoproteins which in turn downregulate the genes that encode for certain cell wall degrading proteins, such as pectin methylesterase, glucan endo-1,3-*β*-D-glucosidase, *β*-1,3-glucanase, endoxyloglucan transferase, pectinesterase, expansin, pectinacetylesterase, *α*-galactosidase, pectate lyase, (1-4)-*β*-mannan endohydrolase, and *β*-galactosidase [10]. Therefore, suppression of *β*-Hex activity in transgenic fruits not only inhibited *N*-glycoprotein degradation but also affects cellulose, hemicellulose, and pectin degradation. Altogether, our phylogenetic analysis of various GH20 *β*-Hexosaminidases with their comparative functional properties suggests that plant *β*-Hexosaminidases are cell wall bound enzymes derived from common bacterial ancestor through multiple gene duplications and are involved in *N*-glycan degradation or processing.

### 3.3. Resolved Predicted 3D Structure and Function

**Figure 4:**
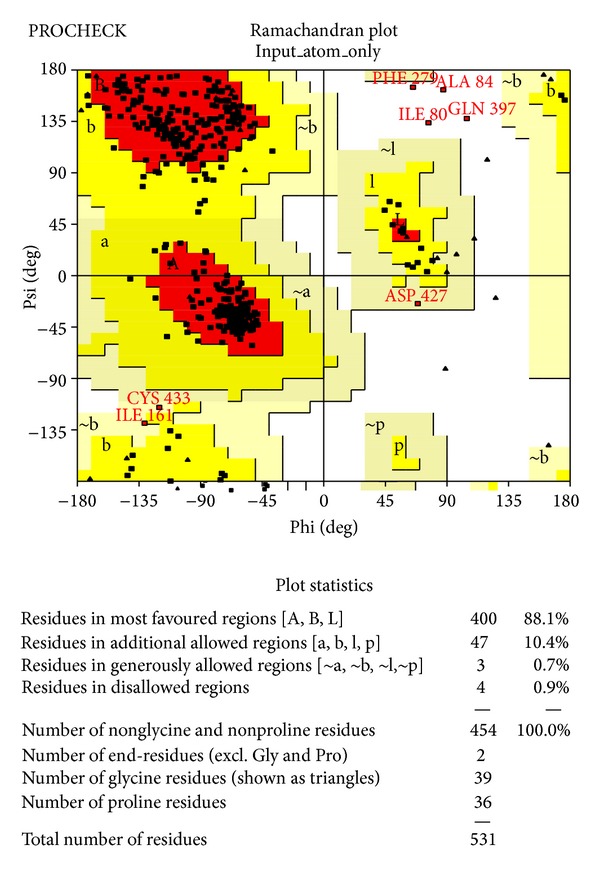
Ramachandran plot of the modeled structure of tomato *β*-*N*-acetyl hexosaminidase provided by PROCHECK.

**Figure 5:**
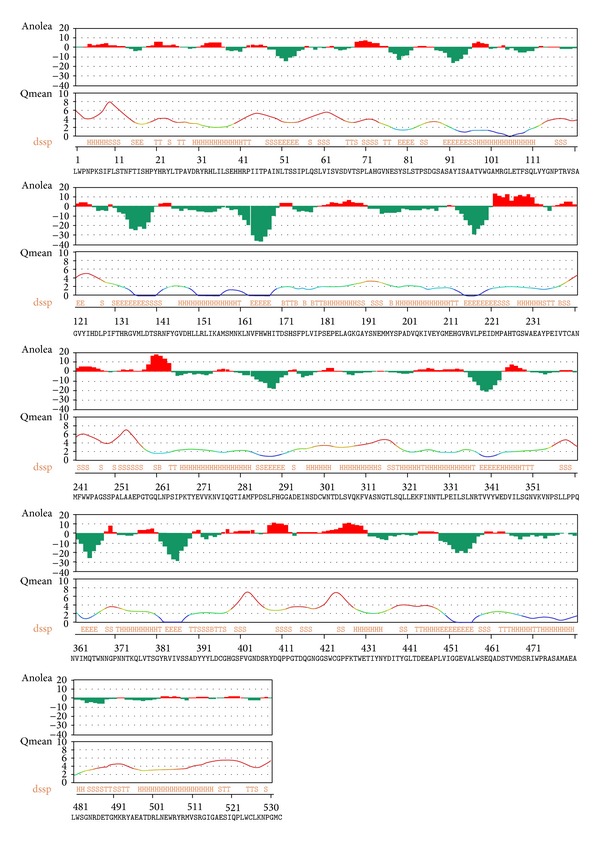
Anolea, Qmean, and DSSP (define secondary structure of protein) obtained from the structural assessment by SWISS-MODEL workplace online software.

The SWISS-MODEL web server [29] was used to identify the 1now as template structure for homology modeling with 38.41% the target-template sequence identity. Another online server ModWeb Comparative Modeling Server version SVN.r1340:1348 M and I-TASSER [30] were also used for further modeling for appropriate model selection. To obtain an accurate homology model, it is very important that appropriate steps are built into the process to assess the quality of the model. Therefore, the accuracies of the predicted models were checked through a series of tests such as DFire [31], QMEAN [32], PROCHECK [33], WHAT_CHECK [34], VERIFY_3D [35], and also ModEval Model evaluation server [36]. A high quality predicted model was obtained from ModWeb comparative modeling web server through the analysis of predicted structures when compared with each other. However, the data for the rest of modeled structures are not shown. The Dfire energy and QMEAN score of best model were −716.03 and 0.511, respectively. The Ramachandran plot showed 88.1% of the residues in the most favoured region, 10.4% in the additional allowed region, 0.7% in the generally allowed region, and only 0.9% in the unfavourable region (Figure 4). Ramachandran *Z*-score is −0.669 indicating how well the backbone conformations of all residues are corresponding to the known allowed areas in the Ramachandran plot and within expected ranges for a well-refined structure. None of the individual amino acid residues was in a bad packaging region. The structural average for the second-generation quality control value is within the normal range. All contacts average is −0.484 and *Z*-score is −2.49, which were within the normal ranges. The Anolea, QMean graph and DSSP (define secondary structure of protein) of modeled *β*-Hex-Sl obtained from the structural assessment by Swiss-model workplace are shown in Figure 5.

**Figure 6:**
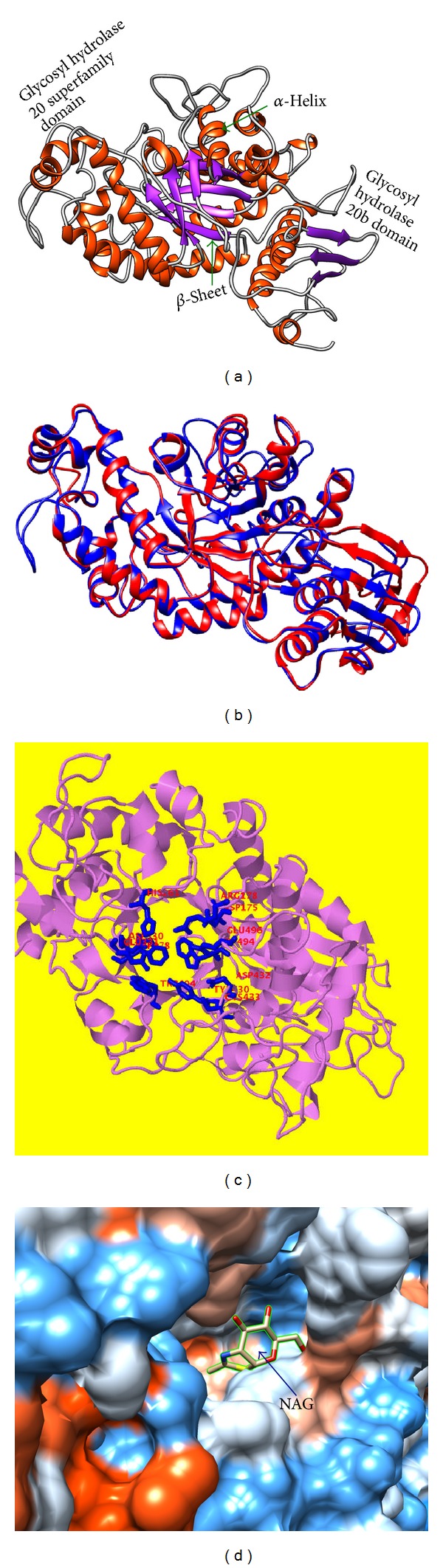
The molecular 3D modeling of tomato* beta*-*N*-acetyl hexosaminidase (*β*-Hex-Sl). SPDB viewer and Chimera were used to prepare the images. (a) The predicted 3D modeled structure is shown as ribbon diagram. The structure contains two fold domains (I and II) including *α*-helix (red), *β*-pleated sheets (purple), and coils (gray) The catalytic domain II is a (*β*/*α*)_8_ barrel with the active site located at the C terminus of the barrel. Template used for building this structure was 1now_B(PDB). (b) Superimposition magic fit image of the modeled structure *β*-Hex-Sl (blue) with template structure human 1now, human *β*-*N*-acetyl-hexosaminidase (red), and human *β*-hexosaminidase B-subunit. (c) The predicted ligand-binding site (active site) residues identified are depicted by as blue color. (d) Space filled view of ligand biding site of *β*-Hex-Sl with docking substrate *N*-acetyl-*β*-D-glucosamine (NAG).

The X-ray crystal structure of human *β*-hexosaminidase started at position 55 of its gene-translated protein sequence [4]. However, the 3D modeled structure of *β*-Hex-Sl started at 38 position of its amino acid sequence as N-terminal. An overall structural model of *β*-Hex-Sl is shown in Figure 6(a), which contains 531 residues in structural parts, glycosyl hydrolase 20b domain-I, and glycosyl hydrolase 20 superfamily domain-II including the (*β*/*α*)_8_ barrel in the middle part. The (*β*/*α*)_8_ barrel structure houses the active site within loops extending from the C termini of the strands that constitute the *β*-barrel. The homologous domains are found in the crystal structure of* S. plicatus* (SpHEX) and* S. marcescens* (SmCHB) [16, 17]. An important secondary-structural motif comprised 19 helices and 13 strands. The *α*- and *β*-contents of the modeled protein were found to be 33.3% and 12.2%, respectively, as predicted by the program PROMOTIF (Figure 6(a)). Structural similarity was further compared by superimposition of modeled structure with template. The modeled structure *β*-Hex-Sl closely resembled the template structure (1nowB) and it had good similarity with the template upon superimposition (Figure 6(b)). The online 3D ligand site prediction software [52] was used to identify the ligand-binding site of the modeled structure *β*-Hex-Sl. The amino acid residues Arg(178), Asp(207), His(261), Asp(330), Glu(331), Trp(378), Trp(404), Tyr(430), Asp(432), Trp(494), and Glu(496) were predicted to be present in the ligand-biding site of *β*-Hex-Sl modeled structure (Figure 6(c)). The space filled view of ligand-biding site of *β*-Hex-Sl with docking substrate *N*-acetyl-*β*-D-glucosamine (NAG) is shown in Figure 6(d). The COFACTOR online software was used to identify the functional motifs including ligand-binding site, gene-ontology terms, and enzyme classification. The top 10 structural analogs of *β*-Hex-Sl modeled structure were identified in the protein data bank (Table 2). The 1nowB, which had the TM-score 0.785 and RMSD 2.21, was found to be the top ranked among the various the homologous proteins analyzed (Table 2). The results indicated that our predicted model structure of *β*-Hex-Sl was good, accurate, and reliable.

The COFACTOR identified *β*-Hex-Sl with the classification EC3.2.1.52 and predicted that amino acid residues Asp(330) and Glu(331) could play important role in enzymatic reaction (Table 3). It was also used to search other known homologous binding to compare the consensus binding with predicted ligand binding site. The three proteins (3lmyA, 2gk1G, 2gjx1) were found to have similar consensus binding sites that were identical to the previously predicted ligand-binding sites (Table 4). To predict the functions of modeled structure of *β*-Hex-Sl, we used COFACTOR and identified 19 gene ontology (GO) terms. The consensus prediction of GO terms and their GO-scores are shown in Table 5. Table 5 shows a consistence of function (GO terms) amongst top scoring templates. The GO score associated with each prediction is defined as the average weight of the GO term, where the weights are assigned based on Cscore^GO^ of the template from which the GO term is derived. The most striking features for *β*-Hex-Sl described by GO terms are homodimerization activities and localization in cell membrane. In humans, two major *β*-hexosaminidase isoenzymes exist: Hex A and Hex B. Hex A is a heterodimer of subunits *α* and *β* (60% identity), whereas Hex B is a homodimer of *β* subunits [4]. The molecular weight of purified *β*-Hex-Sl as determined by gel-filtration (native condition) also showed about four times greater value than that determined by SDS-PAGE (denaturation condition) [10]. This happened due to the dissociation of four subunits from each other by denaturing agent like SDS. The *β*-Hex-Sl modeled 3D structure is a single chain protein containing 531 amino acids but it does not have any other-Hex-subunit like animals. Taken altogether our studies suggested that *β*-Hex-Sl may need to exist as a homotetrameric structure during its functional state and be located at the plant cell wall. Although an involvement of *β*-Hex-Sl in plant cell wall or fruit ripening has been reported recently [10], depending on the properties and behaviour of hexosaminidase homologues we could not exclude the possibilities of their involvements in the other physiological processes such as pathogenic resistance and abiotic stress tolerance in plants.

## 4. Conclusion

We used the 23 previously characterized *β*-hexosaminidases and the 60 novel putative *β*-hexosaminidase amino acid sequences to reconstruct the phylogenetic tree. Phylogenetic analysis placed *β*-Hex-Sl into the plant group, which might originate from the common bacterial ancestral origin by multiple gene duplications. Predicted 3D structure of *β*-Hex-Sl contains 531 amino acids with glycosyl hydrolase 20b domain-I and glycosyl hydrolase 20 superfamily domain-II including the barrel (*β*/*α*)_8_ in the central part. An important secondary-structural motif comprised 19 helices and 13 strands. The *α*- and *β*-contents of the modeled protein were found to be 33.3% and 12.2%, respectively. Eleven amino acids were found to be involved in ligand-binding site of *β*-Hex-Sl. The amino acid residues Asp(330) and Glu(331) could play important role in enzyme-catalyzed reaction. The fully functional state of *β*-Hex-Sl needs to exist as a tetrameric structure and be located at the plant cell wall. The predicted model provides a structural framework that can act as a guide to develop a functional hypothesis to interpret experimental data of *β*-*N*-acetyl-D-hexosaminidases. They may also facilitate efforts to design further site-directed mutagenesis to explore the ligand recognition and the downstream signaling mechanisms for the fruit ripening. The presented modeling approach can be extended to other proteins as well.

## Figures and Tables

**Table 1:** Proteins sequences used for construction of phylogenetic studies.

SL	GI number	Name used in the tree	Description	Organism	Taxonomy
1.	4261632	Homo sapiens-A	***beta*-Hexosaminidase subunit-A, HexA**	*Homo sapiens *	Eukaryota (Primates)
2.	426379627	Gorilla gorilla gorilla	*beta*-Hexosaminidase subunit alpha isoform 1	*Gorilla gorilla gorilla *	//
3.	329112561	Pongo abelii-A	Predicted *beta*-hexosaminidase Subunit-A	*Pongo abelii *	//
4.	332844225	Pan troglodytes-A	*beta*-Hexosaminidase Subunit-A isoform_8	*Pan troglodytes *	//
5.	387849165	Macaca mulatta-A	*beta*-Hexosaminidase Subunit-A precursor	*Macaca mulatta *	//
6.	402874775	Papio anubis-A	*beta*-Hexosaminidase Subunit-A isoform_1	*Papio anubis *	//
7.	635134633	Chlorocebus sabaeus-A	*beta*-Hexosaminidase subunit alpha isoform X5	*Chlorocebus sabaeus *	//
8.	296213630	Callithrix jacchus-A	*beta*-Hexosaminidase Subunit-A isoform_1	*Callithrix jacchus *	//
9.	640780361	Tarsius syrichta-A	*beta*-Hexosaminidase subunit-A isoform X1	*Tarsius syrichta *	//
10.	441617200	Nomascus leucogenys-A	Predicted *beta*-hexosaminidase subunit-A	*Nomascus leucogenys *	//
11.	867691	Homo sapiens-B	***beta*-Hexosaminidase subunit-B, HexB**	*Homo sapiens *	//
12.	114599673	Pan troglodytes-B	*beta*-Hexosaminidase subunit *beta* isoform 5	*Pan troglodytes *	//
13.	297675458	Pongo abelii-B	Predicted *beta*-hexosaminidase subunit *beta*	*Pongo abelii *	//
14.	635028815	Chlorocebus sabaeus-B	Predicted *beta*-hexosaminidase subunit *beta*	*Chlorocebus sabaeus *	//
15.	388454685	Macaca mulatta-B	***beta*-Hexosaminidase subunit *beta***	*Macaca mulatta *	//
16.	402871850	Papio Anubis-B	Predicted *beta*-hexosaminidase subunit *beta*	*Papio anubis *	//
17.	296194339	Callithrix jacchus-B	*beta*-Hexosaminidase subunit *beta* isoform 1	*Callithrix jacchus *	//
18.	403256462	Saimiri boliviensis-B	Predicted *beta*-hexosaminidase subunit *beta*	*Saimiri boliviensis *	//
19.	478492476	Ceratotherium simum-B	Predicted *beta*-hexosaminidase subunit *beta*	*Ceratotherium simum *	//
20.	17647501	Drosophila melanogaster-1	***beta*-Hexosaminidase, Hex1**	*Drosophila melanogaster *	Eukaryota (Insect)
21.	557771663	Musca domestica1	*beta*-*N*-Acetylglucosaminidase-like isoform X1	*Musca domestica *	//
22.	498964043	Ceratitis capitata1	*beta*-*N*-Acetylglucosaminidase-like isoform X1	*Ceratitis capitata *	//
23.	498931058	Ceratitis capitata1-1	*beta*-*N*-Acetylglucosaminidase-like isoform X1	*Ceratitis capitata *	//
24.	157106934	Aedes aegypti1	*beta*-Hexosaminidase	*Aedes aegypti *	//
25.	170057261	Culex quinquefasciatus1	*beta*-*N*-Acetylglucosaminidase	*C. quinquefasciatus *	//
26.	508082176	Spodoptera frugiperda	**Lysosomal *beta*-hexosaminidase**	*Spodoptera frugiperda *	//
27.	294988604	Agrotis ipsilon	***beta*-*N*-Acetyl hexosaminidase**	*Agrotis ipsilon *	//
28.	19072855	Trichoplusia ni	***beta*-*N*-Acetyl hexosaminidase**	*Trichoplusia ni *	//
29.	62722476	Choristoneura fumiferana	***beta*-*N*-Acetyl hexosaminidase**	*Choristoneura fumiferana *	//
30.	114842947	Ostrinia furnacalis1	***beta*-*N*-Acetylglucosaminidase**	*Ostrinia furnacalis *	//
31.	37678109	Manduca sexta	***beta*-*N*-Acetylglucosaminidase**	*Manduca sexta *	//
32.	17933586	Drosophila melanogaster-2	***beta*-Hexosaminidase, Hex2**	*Drosophila melanogaster *	//
33.	557764625	Musca domestica2	*beta*-*N*-Acetylglucosaminidase-like	*Musca domestica *	//
34.	499003284	Ceratitis capitata2	*beta*-*N*-Acetylglucosaminidase-like	*Ceratitis capitata *	//
35.	157117066	Aedes aegypti2	***beta*-*N*-Acetyl hexosaminidase**	*Aedes aegypti *	//
36.	642910295	Tribolium castaneum	*beta*-*N*-Acetyl hexosaminidase isoform X1	*Tribolium castaneum *	//
37.	170029661	Culex quinquefasciatus2	*beta*-*N*-Acetylglucosaminidase-like	*C. quinquefasciatus *	//
38.	157804574	Ostrinia furnacalis2	***beta*-*N*-Acetyl hexosaminidase**	*Ostrinia furnacalis *	//
39.	145651816	Bombyx mori	***beta*-*N*-Acetyl hexosaminidase precursor**	*Bombyx mori *	//
40.	350540008	Solanum lycopersicum2	***beta*-Hexosaminidase1**	*Solanum lycopersicum *	Eukaryota (planta)
41.	565386664	Solanum tuberosum2	Predicted *beta*-hexosaminidase 2-like	*Solanum tuberosum *	//
42.	315440799	Capsicum annuum2	***beta*-*N*-Acetylhexosaminidase**	*Capsicum annuum *	//
43.	225450263	Vitis vinifera2	Predicted *beta*-hexosaminidase-like	*Vitis vinifera *	//
44.	449532074	Cucumis sativus2	Predicted *beta*-hexosaminidase 2-like	*Cucumis sativus *	//
45.	255581813	Ricinus communis	Putative *beta*-hexosaminidase	*Ricinus communis *	//
46.	440355382	Prunus persica2	***beta*-Hexosaminidase 2**	*Prunus persica *	//
47	568858509	Citrus sinensis2	Predicted *beta*-hexosaminidase 2-like	*Citrus sinensis *	//
48.	15220590	Arabidopsis thaliana2	***beta*-Hexosaminidase 2**	*Arabidopsis thaliana *	//
49.	568879684	Citrus sinensis3	Predicted *beta*-hexosaminidase 2-like	*Citrus sinensis *	//
50.	356528621	Glycine max2	Predicted *beta*-hexosaminidase 2-like	*Glycine max *	//
51.	357116549	Brachypodium distachyon2	Predicted *beta*-hexosaminidase 2-like	*Brachypodium distachyon *	//
52.	30694211	Arabidopsis thaliana1	***beta*-Hexosaminidase 1**	*Arabidopsis thaliana *	//
53.	567186303	Eutrema salsugineum	Hypothetical Protein	*Eutrema salsugineum *	//
54.	449459940	Cucumis sativus1	Predicted *beta*-hexosaminidase 1-like	*Cucumis sativus *	//
55.	356568953	Glycine max1	Predicted *beta*-hexosaminidase 1-like	*Glycine max *	//
56.	401065909	Prunus persica1	***beta*-Hexosaminidase**	*Prunus persica *	//
57.	565358237	Solanum tuberosum1	Predicted *beta*-hexosaminidase 1-like	*Solanum tuberosum *	//
58.	350538741	Solanum lycopersicum1	Predicted *beta*-hexosaminidase 2	*Solanum lycopersicum *	//
59.	357134815	Brachypodium distachyon1	*beta*-Hexosaminidase subunit- B2-like isoform	*Brachypodium distachyon *	//
60.	573945166	Oryza brachyantha	Predicted *beta*-hexosaminidase 1-like	*Oryza brachyantha *	//
61.	115461737	Oryza sativa	Putative *beta*-hexosaminidase	*Oryza sativa *	//
62.	169766420	Aspergillus oryzae	***beta*-*N*-Acetylglucosaminidase**	*Aspergillus oryzae *	Eukaryota (Fungi)
63.	238483137	Aspergillus flavus	Putative *beta*-*N*-Acetylhexosaminidase	*Aspergillus flavus *	//
64.	115491163	Aspergillus terreus	Putative *beta*-hexosaminidase precursor	*Aspergillus terreus *	//
65.	119484544	Neosartorya fischeri	Putative *beta*-hexosaminidase	*Neosartorya fischeri *	//
66.	145241784	Aspergillus niger	*Predicted N*-acetylglucosaminidase	*Aspergillus niger *	//
67.	70983560	Aspergillus fumigatus	*Predicted beta*-*N*-acetylhexosaminidase	*Aspergillus fumigatus *	//
68.	358375826	Aspergillus kawachii-1	*beta*-*N*-Acetylhexosaminidase	*Aspergillus kawachii *	//
69.	121719823	Aspergillus clavatus	Putative *beta*-*N*-acetylhexosaminidase	*Aspergillus clavatus *	//
70.	358372216	Aspergillus kawachii-2	*beta*-*N*-Acetylhexosaminidase precursor	*Aspergillus kawachii *	//
71.	525585306	Penicillium oxalicum	Putative *beta*-1,6-*N*-acetylglucosaminidase	*Penicillium oxalicum *	//
72.	557727225	Byssochlamys spectabilis	Putative *beta*-*N*-acetylhexosaminidase	*Byssochlamys spectabilis *	//
73.	13786695	Streptomyces Plicatus	***beta*-*N*-Acetylhexosaminidase, SpHex**	*Streptomyces Plicatus *	Prokaryote (Bacteria)
74.	494714113	Streptomyces coelicoflavus	Predicted *beta*-hexosaminidase	*Streptomyces coelicoflavus *	//
75.	511095822	Streptomyces lividans	Putative *beta*-hexosaminidase precursor	*Streptomyces lividans *	//
76.	490099150	Streptomyces viridochromogenes1	Putative *beta*-hexosaminidase	*Streptomyces viridochromogenes *	//
77.	499338878	Streptomyces coelicolor1	Putative *beta*-hexosaminidase	*Streptomyces coelicolor *	//
78.	640930344	Streptomyces olindensis	Predicted *beta*-hexosaminidase	*Streptomyces olindensis *	//
79.	493092893	Streptomyces gancidicus	Predicted *beta*-hexosaminidase	*S. gancidicus *	//
80.	594145706	Streptomyces coelicolor2	***beta*-Hexosaminidase**	*Streptomyces Coelicolor *	//
81.	505473521	Streptomyces davawensis	*beta*-*N*-Acetylhexosaminidase	*Streptomyces davawensis *	//
82.	490088482	Streptomyces viridochromogenes2	*beta*-*N*-Acetylhexosaminidase	*Streptomyces viridochromogenes *	//
83.	119720203	Thermofilum pendens	Glycoside hydrolase family protein	*Thermofilum pendens *	Archea

Bold font indicates the experimentally characterized *beta*-*N*-acetylhexosaminidases.

**Table 2:** Top 10 identified structural analogs in PDB by COFACTOR.

Rank	PDB Hit	TM-score	RMSD^a^	IDEN^a^	Cov.
1	1nowB	0.785	2.21	0.339	0.823
2	2gjxH	0.777	2.60	0.298	0.827
3	3s6tA	0.769	3.07	0.297	0.844
4	1c7sA	0.751	3.66	0.198	0.848
5	3rcnA	0.723	3.76	0.230	0.815
6	4h04A	0.709	4.33	0.168	0.842
7	3gh7A	0.707	3.63	0.244	0.795
8	1hp5A	0.701	3.47	0.236	0.783
9	2eplX	0.671	3.89	0.120	0.787
10	1qba_3	0.566	3.18	0.236	0.622

TM-score is a measure of global structural similarity between query and template protein.

RMSD^a^ is the RMSD between residues that are structurally aligned by TM-align.

IDEN^a^ is the percentage sequence identity in the structurally aligned region.

Cov. represents the coverage of the alignment by TM-align and is equal to the number of structurally aligned residues divided by length of the query protein.

**Table 3:** Top 5 enzyme homologs in PDB by COFACTOR.

Rank	Cscore^EC^	PDB Hit	TM-score	RMSD	IDEN	Cov	EC number	Predicted active site residues
1	0.576	2gjxA	0.776	2.53	0.300	0.825	3.2.1.52	330, 331
2	0.512	1hp4A	0.698	3.47	0.236	0.781	3.2.1.52	330, 331
3	0.508	3gh4A	0.706	3.64	0.244	0.795	3.2.1.52	330, 331
4	0.173	1o7aA	0.784	2.34	0.338	0.825	3.2.1.52	330, 331
5	0.142	1yhtA	0.502	3.60	0.166	0.565	3.2.1.52	330, 331

Cscore^EC^ is the confidence score for the enzyme classification (EC) number prediction. Cscore^EC^ values range in between [0-1], where a higher score indicates a more reliable EC number prediction.

TM-score is a measure of global structural similarity between query and template protein.

RMSD^a^ is the RMSD between residues that are structurally aligned by TM-align.

IDEN^a^ is the percentage sequence identity in the structurally aligned region.

Cov. represents the coverage of global structural alignment and is equal to the number of structurally aligned residues divided by length of the query protein.

**Table 4:** Template proteins with similar binding sites searched by COFACTOR.

Rank	Cscore^LB^	PDB Hit	TM-score	RMSD^a^	IDEN^a^	Cov.	BS-score	Lig. Name	Predicted binding sites
1	0.64	3lmyA	0.78	2.19	0.345	0.82	1.55	CP6	178, 204, 207, 261, 330, 404, 430, 432, 433, 494, 496
2	0.45	2gk1G	0.77	2.56	0.300	0.82	1.50	NGT	178, 251, 330, 331, 378, 404, 429, 494, 496
3	0.06	2gjx1	0.78	2.56	0.344	0.82	0.95	Peptide	178, 179, 227, 228, 230, 231, 464, 496, 497, 499, 500, 501, 502, 505, 506

Cscore^LB^ is the confidence score of predicted binding site. Cscore^LB^ values range in between [0-1], where a higher score is better site prediction.

BS-score is a measure of local similarity (sequence and structure) between template binding site and predicted binding site in the query structure. Based on large scale benchmarking analysis; we have observed that a BS-score > 1 reflects a significant local match between the predicted and template binding site.

TM-score is a measure of global structural similarity between query and template protein.

RMSD^a^ is the RMSD between residues that are structurally aligned by TM-align.

IDEN^a^ is the percentage sequence identity in the structurally aligned region.

Cov. represents the coverage of global structural alignment and is equal to the number of structurally aligned residues.

**Table 5:** Consensus prediction of gene ontology terms searched by COFACTOR.

Molecular function		Biological process		Cellular function	
GO term	GO score	GO term	GO score	GO term	GO score
GO:0043169	0.96	GO:0006689	0.80	GO:0016020	0.80
GO:0046982	0.80	GO:0030203	0.80	GO:0005764	0.80
GO:0005529	0.56	GO:0042552	0.80	GO:0005625	0.56
GO:0016231	0.56	GO:0050885	0.80	GO:0001669	0.56
GO:0042803	0.56	GO:0019915	0.80		
		GO:0007605	0.80		
		GO:0007040	0.80		
		GO:0001501	0.80		
		GO:0008219	0.80		
		GO:0031323	0.56		

Table 5 shows a consistence of function (GO terms) amongst top scoring templates. The GO score associated with each prediction is defined as the average weight of the GO term, where the weights are assigned based on Cscore^GO^ of the template from which the GO term is derived.

## References

[1] Hossain MA, Nakano R, Nakamura K, Kimura Y (2010). Molecular identification and characterization of an acidic peptide:*N*-glycanase from tomato *(Lycopersicum esculentum*) fruits. *Journal of Biochemistry*.

[2] Nakamura K, Inoue M, Maeda M (2009). Molecular cloning and gene expression analysis of tomato endo-*β*-n-acetylglucosaminidase, an endoglycosidase involved in the production of high-mannose type free N-glycans during tomato fruit ripening. *Bioscience, Biotechnology and Biochemistry*.

[3] Hossain MA, Nakano R, Nakamura K, Hossain MT, Kimura Y (2010). Molecular characterization of plant acidic *α*-mannosidase, a member of glycosylhydrolase family 38, involved in the turnover of *N*-glycans during tomato fruit ripening. *Journal of Biochemistry*.

[4] Mark BL, Mahuran DJ, Cherney MM, Zhao D, Knapp S, James MNG (2003). Crystal structure of human *β*-hexosaminidase *β*: understanding the molecular basis of sandhoff and tay-sachs
disease. *Journal of Molecular Biology*.

[5] Park HC, Hwang JH, Kang A-Y (2012). Urinary N-acetyl-*β*-D glucosaminidase as a surrogate marker for renal function in autosomal dominant polycystic kidney disease: 1 year prospective cohort study. *BMC Nephrology*.

[6] Liu T, Zhang H, Liu F, Wu Q, Shen X, Yang Q (2011). Structural determinants of an insect *β*-N-acetyl-D-hexosaminidase specialized as a chitinolytic enzyme. *Journal of Biological Chemistry*.

[7] Miranda PV, González-Echeverría F, Blaquier JA, Mahuran DJ, Tezón JG (2000). Evidence for the participation of *β*-hexosaminidase in human sperm-zona pellucida interaction in vitro. *Molecular Human Reproduction*.

[8] Cattaneo F, Intra J, Matsumoto M, Briani F, Hoshi M, Perotti ME (2006). Identification and expression analysis of Drosophila melanogaster genes encoding *β*-hexosaminidases of the sperm plasma membrane. *Glycobiology*.

[9] Vaněk O, Brynda J, Hofbauerová K (2011). Crystallization and diffraction analysis of *β*-N-acetylhexosaminidase from Aspergillus oryzae. *Acta Crystallographica F: Structural Biology and Crystallization Communications*.

[10] Meli VS, Ghosh S, Prabha TN, Chakraborty N, Chakraborty S, Datta A (2010). Enhancement of fruit shelf life by suppressing *N*-glycan processing enzymes. *Proceedings of the National Academy of Sciences of the United States of America*.

[11] Strasser R, Bondili JS, Schoberer J (2007). Enzymatic properties and subcellular localization of arabidopsis *β*-N-acetylhexosaminidases. *Plant Physiology*.

[12] Jagadeesh BH, Prabha TN, Srinivasan K (2004). Activities of *β*-hexosaminidase and *α*-mannosidase during development and ripening of bell capsicum (*Capsicum annuum* var. variata). *Plant Science*.

[13] Jin YL, Jo YY, Kim KY, Shim JH, Kim YW, Park RD (2002). Purification and characterization of *β*-*N*-acetylhexosaminidase from rice seeds.. *Journal of biochemistry and molecular biology*.

[14] Maier T, Strater N, Schuette CG, Klingenstein R, Sandhoff K, Saenger W (2003). The X-ray crystal structure of human *β*-hexosaminidase B provides new insights into Sandhoff disease. *Journal of Molecular Biology*.

[15] Mark BL, Vocadlo DJ, Knapp S, Triggs-Raine BL, Withers SG, James MNG (2001). Crystallographic evidence for substrate-assisted catalysis in a bacterial *β*-hexosaminidase. *The Journal of Biological Chemistry*.

[16] Williams SJ, Mark BL, Vocadlo DJ, James MNG, Withers SG (2002). Aspartate 313 in the *Streptomyces plicatus* hexosaminidase plays a critical role in substrate-assisted catalysis by orienting the 2-acetamido group and stabilizing the transition state. *Journal of Biological Chemistry*.

[17] Prag G, Papanikolau Y, Tavlas G, Vorgias CE, Petratos K, Oppenheim AB (2000). Structures of chitobiase mutants complexed with the substrate di-*N*-acetyl-D-glucosamine: the catalytic role of the conserved acidic pair, aspartate 539 and glutamate 540. *Journal of Molecular Biology*.

[18] Ramasubbu N, Thomas LM, Ragunath C, Kaplan JB (2005). Structural analysis of dispersin B, a biofilm-releasing glycoside hydrolase from the periodontopathogen Actinobacillus actinomycetemcomitans. *Journal of Molecular Biology*.

[19] Sumida T, Ishii R, Yanagisawa T, Yokoyama S, Ito M (2009). Molecular cloning and crystal structural analysis of a novel *β*-*N*- acetylhexosaminidase from *Paenibacillus* sp. TS12 capable of degrading glycosphingolipids. *Journal of Molecular Biology*.

[20] Langley DB, Harty DWS, Jacques NA, Hunter N, Guss JM, Collyer CA (2008). Structure of N-acetyl-*β*-D-glucosaminidase (GcnA) from the endocarditis pathogen *Streptococcus gordonii* and its complex with the mechanism-based inhibitor NAG-thiazoline. *Journal of Molecular Biology*.

[21] Jiang YL, Yu WL, Zhang JW (2011). Structural basis for the substrate specificity of a novel *β*-N-acetylhexosaminidase StrH protein from *Streptococcus pneumoniae* R6. *The Journal of Biological Chemistry*.

[22] Gonzalez DS, Jordan IK (2000). The *α*-mannosidases: phylogeny and adaptive diversification. *Molecular Biology and Evolution*.

[23] Huang Y, Niu B, Gao Y, Fu L, Li W (2010). CD-HIT Suite: a web server for clustering and comparing biological sequences. *Bioinformatics*.

[24] Wheeler DL, Barrett T, Benson DA (2007). Database resources of the National Center for Biotechnology Information. *Nucleic Acids Research*.

[25] Edgar RC (2004). MUSCLE: a multiple sequence alignment method with reduced time and space complexity. *BMC Bioinformatics*.

[26] Castresana J (2000). Selection of conserved blocks from multiple alignments for their use in phylogenetic analysis. *Molecular Biology and Evolution*.

[27] Felsenstein J (2000). *PHYLIP: Phylogeny Inference Package, Version 3.6 (Alpha)*.

[28] Whelan S, Goldman N (2001). A general empirical model of protein evolution derived from multiple protein families using a maximum-likelihood approach. *Molecular Biology and Evolution*.

[29] Arnold K, Bordoli L, Kopp J, Schwede T (2006). The SWISS-MODEL workspace: a web-based environment for protein structure homology modelling. *Bioinformatics*.

[30] Zhang Y (2008). I-TASSER server for protein 3D structure prediction. *BMC Bioinformatics*.

[31] Zhou H, Zhou Y (2002). Distance-scaled, finite ideal-gas reference state improves structure-derived potentials of mean force for structure selection and stability prediction. *Protein Science*.

[32] Benkert P, Biasini M, Schwede T (2011). Toward the estimation of the absolute quality of individual protein structure models. *Bioinformatics*.

[33] Laskowski RA, MacArthur MW, Moss DS, Thornton JM (1993). PROCHECK: a program to check the stereochemical quality of protein structures. *Journal of Applied Crystallography*.

[34] Hooft RWW, Vriend G, Sander C, Abola EE (1996). Errors in protein structures. *Nature*.

[35] Luthy R, Bowie JU, Eisenberg D (1992). Assesment of protein models with three-dimensional profiles. *Nature*.

[36] Melo F, Sánchez R, Sali A (2002). Statistical potentials for fold assessment. *Protein Science*.

[37] Roy A, Yang J, Zhang Y (2012). COFACTOR: an accurate comparative algorithm for structure-based protein function annotation. *Nucleic Acids Research*.

[38] Roy A, Zhang Y (2012). Recognizing protein-ligand binding sites by global structural alignment and local geometry refinement. *Structure*.

[39] Yang J, Roy A, Zhang Y (2013). BioLiP: a semi-manually curated database for biologically relevant ligand-protein interactions. *Nucleic Acids Research*.

[40] Marques-Bonet T, Girirajan S, Eichler EE (2009). The origins and impact of primate segmental duplications. *Trends in Genetics*.

[41] Zhou X, Lin Z, Ma H (2010). Phylogenetic detection of numerous gene duplications shared by animals, fungi and plants. *Genome Biology*.

[42] Zwierz K, Zalewska A, Zoch-Zwierz W (1999). Isoenzymes of N-acetyl-*β*-hexosaminidase. *Acta Biochimica Polonica*.

[43] Fukuda T, Yokoyama J, Nakamura T (2005). Molecular phylogeny and evolution of alcohol dehydrogenase (Adh) genes in legumes. *BMC Plant Biology*.

[44] Bulawa CE (1993). Genetics and molecular biology of chitin synthesis in fungi. *Annual Review of Microbiology*.

[45] Barber MS, Ride JP (1989). Purification and properties of a wheat leaf N-acetyl-*β*-d-hexosaminidase. *Plant Science*.

[46] Oikawa A, Itoh E, Ishihara A, Iwamura H (2003). Purification and characterization of *β*-*N*-acetylhexosaminidase from maize seedlings. *Journal of Plant Physiology*.

[47] Harris N, Chrispeels M (1975). Histochemical and biochemical observations on storage protein metabolism and protein body autolysis in cotyledons of germinating mung beans. *Plant Physiology*.

[48] Dowd PF, Johnson ET, Pinkerton TS (2007). Oral toxicity of *β*-*N*-acetyl hexosaminidase to insects. *Journal of Agricultural and Food Chemistry*.

[49] Jagadeesh BH, Prabha TN, Srinivasan K (2004). Activities of glycosidases during fruit development and ripening of tomato (*Lycopersicum esculantum* L.): implication in fruit ripening. *Plant Science*.

[50] Hossain MA, Ranam MM, Kimura Y, Roslan HA Changes in biochemical Character istics and activities of ripening associated enzymes in mango fruit during storage at different temperatures.

[51] Ghosh S, Meli VS, Kumar A (2011). The *N*-glycan processing enzymes *α*-mannosidase and *β*-D-*N*-acetylhexosaminidase are involved in ripening-associated softening in the non-climacteric fruits of capsicum. *Journal of Experimental Botany*.

[52] Wass MN, Kelley LA, Sternberg MJE (2010). 3DLigandSite: predicting ligand-binding sites using similar structures. *Nucleic Acids Research*.

